# A growing heart: a literary review on clozapine-induced Myocarditis

**DOI:** 10.1192/j.eurpsy.2022.674

**Published:** 2022-09-01

**Authors:** F. Ramalheira, M. Conde Moreno, A. Vieira, B. Freitas, M.D.C. Vasconcelos

**Affiliations:** 1 Centro hospitalar Psiquiátrico de Lisboa, Serviço De Electroconvulsoterapia, Lisboa, Portugal; 2 Centro hospitalar Psiquiátrico de Lisboa, Hospital De Dia, Lisboa, Portugal; 3 Centro Hospitalar Psiquiátrico de Lisboa, Ccsmo, Lisbon, Portugal; 4 Centro Hospitalar Psiquiátrico de Lisboa, Psiquiatria, Lisboa, Portugal

**Keywords:** clozapine-induced myocarditis, clozapine, myocarditis

## Abstract

**Introduction:**

Clozapine, a unique antipsychotics, is well known for its adverse effects. Myocarditis is a rare but life-threatening complication, however not monitored at a global scale.

**Objectives:**

This work aims to review the literature on clozapine-induced myocarditis.

**Methods:**

Pubmed and Google Scholar search using Mesh terms clozapine, myocarditis, clozapine-induced myocarditis.

**Results:**

Clozapine-induced Myocarditis (CIM) is potentially fatal, with mortality rates environ 21%. According to the World Health Organization Monitoring Program, notification rate is 0,93%, nonetheless incidence found in literature varies dramatically. Highest rates are reported in Australia, where this relationship was first established and a complete monitoring protocol is compulsory in all patients starting clozapine, which causes some authors to defend this condition is generally undernotified. Underlying mechanisms are not fully understood, but an imunomediated hypersensitive reaction occurring in the first 3-4 weeks after treatment is suggested. CIM is rare after 6 weeks. Risk factors include age, cardiac disease, initial high dose, rapid titration and simultaneous valproate or other antipsychotics use. The most common symptoms, fever, tachycardia, dyspnea and malaise, are non-specific and can be indistinguishable from other clozapine benign adverse effects. Analytically, C-reactive protein and Troponine elevation are the most specific diagnostic markers, therefore the most suitable for monitoring. Prompt cardiological observation for further evaluation should be seeked whenever CIM is suspected.

**Conclusions:**

Diagnosis of CIM can be challenging. Systematic monitoring is not consensual but may increase detection, prevent severe outcomes and help clinicians decide whether to keep or suspend therapy. Clozapine is beneficial and shouldn’t be avoided or unjustifiably discontinued.

**Disclosure:**

No significant relationships.

